# Real-World Assessment of Osteoporosis-Related Polymorphisms: Negative Findings for Osteoporosis and an Exploratory Association with Vitamin D

**DOI:** 10.3390/life16020259

**Published:** 2026-02-03

**Authors:** Kyung-Won Hong, Myungshin Kim, Inseok Lee, Ja-Eun Choi, Hyun-Young Shin

**Affiliations:** 1 Theragen Health Co., Ltd., Seongnam-si 13493, Gyeonggi-do, Republic of Korea; kyungwon.hong@theragenhealth.com (K.-W.H.); jaeun.choi@theragenhealth.com (J.-E.C.); 2 Department of Laboratory Medicine, College of Medicine, The Catholic University of Korea, Seoul 06591, Republic of Korea; microkim@catholic.ac.kr; 3 Department of Internal Medicine, College of Medicine, The Catholic University of Korea, Seoul 06591, Republic of Korea; isle@catholic.ac.kr; 4 Department of Family Medicine, Seoul St. Mary’s Hospital, College of Medicine, The Catholic University of Korea, Seoul 06591, Republic of Korea

**Keywords:** osteoporosis, vitamin D, polymorphism, association

## Abstract

Osteoporosis develops silently and is strongly influenced by both genetic and environmental factors. This study investigated whether three well-established osteoporosis-related polymorphisms—*SOST* rs1513670, *LRP5* rs3736228, and *ESR1* rs6929137—derived from a commercial genetic testing panel (HelloGene™) were primarily associated with osteoporosis prevalence and/or incidence and secondarily with bone-related biochemical markers in a Korean population. A total of 843 adults who completed genetic testing at Seoul St. Mary’s Hospital and subsequently underwent bone mineral density (BMD) assessment were included. Logistic and linear regression models were applied to evaluate associations between genotypes, osteoporosis diagnosis, and serum calcium and 25-hydroxyvitamin D levels. None of the examined SNPs showed significant associations with osteoporosis status. However, the SOST variant demonstrated a statistically significant association with serum vitamin D concentration (β = −4.836, *p* = 1.7 × 10^−6^), with TC and CC genotype carriers exhibiting markedly lower vitamin D levels than TT carriers. LRP5 and ESR1 variants showed no significant relationships with either osteoporosis or vitamin D. These findings suggest a hypothesis-generating finding between SOST-mediated WNT signaling and vitamin D metabolism, even in the absence of measurable effects on clinical osteoporosis.

## 1. Introduction

Osteoporosis is a skeletal disorder characterized by progressive deterioration of bone microarchitecture, resulting in increased fragility and susceptibility to fractures following minimal mechanical stress [1,2]. Because early-stage osteoporosis is typically asymptomatic, the disease often progresses silently until a fracture occurs, leading to substantial morbidity and mortality. Low bone mineral density (BMD) affects more than 200 million individuals worldwide and imposes a significant global health burden, accounting for an estimated 8.32 million years lived with disability (YLDs), 17.2 million disability-adjusted life years (DALYs), and approximately 477,000 deaths globally in 2020 among adults aged 40 years and older [3]. Genetic factors are known to play a dominant role in osteoporosis susceptibility, contributing to nearly 80% of disease incidence [1,4,5].

Vitamin D is a critical regulator of skeletal health, maintaining calcium and phosphate homeostasis and facilitating intestinal calcium absorption required for proper bone mineralization [6]. Vitamin D deficiency induces secondary hyperparathyroidism, accelerates bone turnover, and contributes to progressive declines in BMD, thereby increasing the risk of osteoporosis and fragility fractures [7]. Large-scale epidemiological studies consistently demonstrate that low serum 25-hydroxyvitamin D [25(OH)D] concentrations are associated with a higher prevalence of osteopenia and osteoporosis, particularly among older adults and postmenopausal women [8]. In addition, inadequate vitamin D status adversely affects neuromuscular function, increasing fall risk and further exacerbating fracture susceptibility [9]. Collectively, these findings underscore vitamin D as a pivotal and modifiable determinant of bone health and fracture prevention [10].

Vitamin D deficiency is particularly prevalent in the Korean population, largely attributable to limited sun exposure, indoor-oriented lifestyles, and pronounced seasonal variation [11]. National health surveys indicate that a substantial proportion of Korean adults—including younger individuals—exhibit serum 25(OH)D levels below recommended thresholds for optimal bone health [12]. Consequently, Korean populations may face an elevated risk of osteopenia, osteoporosis, and related fractures, highlighting the importance of improved monitoring strategies and targeted interventions to address vitamin D insufficiency and protect skeletal integrity [13].

In addition to environmental and nutritional influences, genetic predisposition plays a central role in determining osteoporosis risk [14]. Since the mid-2000s, genetic studies have identified multiple biological pathways involved in BMD regulation and fracture susceptibility, among which the WNT signaling pathway has emerged as a key regulator of bone formation and remodeling [15,16]. Hormonal signaling, particularly through estrogen receptors, also contributes significantly to bone homeostasis, especially in women after menopause [17]. Building on these established biological mechanisms, there is growing interest in assessing whether genetic information derived from commercial prediction panels can meaningfully reflect osteoporosis-related phenotypes in real-world health examination settings. In this study, we evaluated a three-SNP osteoporosis panel comprising two WNT pathway-related variants—low-density lipoprotein receptor-related protein 5 (LRP5; rs3736228) and sclerostin (SOST; rs1513670)—and an estrogen receptor polymorphism (ESR1; rs6929137), all of which have been frequently implicated in BMD variation and osteoporosis risk (Table 1) [16,17]. The primary hypothesis was that this three-SNP panel would be associated with osteoporosis status as defined by DXA-based criteria or predefined phenotypes in a routine health examination context. As a secondary, exploratory objective, we examined associations between these genetic variants and serum 25(OH)D concentrations, calcium levels, and other biochemical markers relevant to bone metabolism.

## 2. Materials and Methods

### 2.1. Study Population

**Figure 1:**
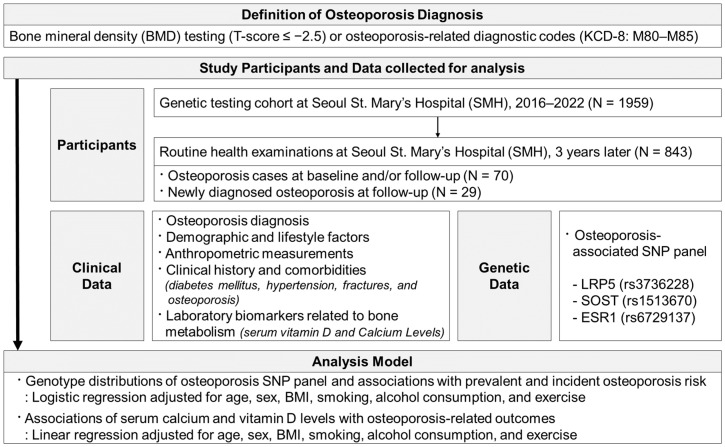
Schematic overview of the study design.

Participants were individuals who voluntarily underwent a commercial genetic testing service (HelloGene™ version 3.0 or 4.0, Theragen Health Co., Ltd., Seongnam, Republic of Korea) as part of routine health examinations conducted between 2016 and February 2022 at Seoul St. Mary’s Hospital (SMH), located in the southern region of Seoul, Korea. Individuals were eligible for inclusion if they completed the genetic test at baseline and subsequently underwent bone mineral density (BMD) assessment during follow-up. Participants with a prior clinical diagnosis of osteoporosis or those receiving osteoporosis-related pharmacologic treatment at baseline were excluded (Figure 1).

Among individuals with available follow-up data, those who had been monitored at SMH for at least three years after genetic testing were included in the analysis. Osteoporosis cases were defined as participants who either (1) received a diagnosis of osteoporosis based on BMD testing or (2) were assigned an osteoporosis-related diagnostic code (KCD-8: M80–M85) at SMH during follow-up. Only individuals who had previously provided written informed consent for genetic testing as part of routine health examinations were included. The health data of patients who visited St. Mary’s Hospital is utilized for research purposes only with the consent of those who agreed initially. All genetic and clinical data used in this study were fully anonymized prior to analysis. This study was conducted in accordance with the principles of the Declaration of Helsinki and was approved by the Institutional Review Board (IRB) of Seoul St. Mary’s Hospital (IRB date: 8 July 2025, Approval No. KIRB-20250708-004).

### 2.2. Bone Mineral Density Measurement and Osteoporosis Definition

Bone mineral density (BMD) was measured using dual-energy X-ray absorptiometry (DXA) with a GE Lunar Prodigy system [24]. BMD assessments were obtained from both the lumbar spine and bilateral femoral neck regions [24]. Osteoporosis was defined as a T-score ≤ −2.5 at either measurement site, in accordance with established diagnostic criteria.

The DXA equipment demonstrated excellent scan-to-scan repeatability, with a coefficient of variation of approximately ±0.5 percentage points, ensuring high measurement precision [24]. To minimize inter-machine variability, follow-up BMD assessments were performed using the same DXA system whenever possible [24].

### 2.3. Key Variables

Participants completed standardized self-administered questionnaires at the time of health screening, which collected information on age, sex, smoking habits, alcohol consumption, and regular exercise status. Routine anthropometric measurements—including height, weight, and body mass index (BMI)—were obtained by trained medical staff.

Clinical histories were extracted from electronic medical records and included major comorbidities such as diabetes mellitus (KCD-8 codes: E10–E14, E28, E36), hypertension (I10–I14, I67.4, or self-reported history of hypertension on the health survey), osteoporosis, and bone fractures (M84.0–M84.4).

Laboratory biomarkers relevant to bone metabolism were measured during the health examination, including serum calcium and 25-hydroxyvitamin D [25(OH)D] concentrations. The measurement of 25(OH) Vitamin D is performed in the hospital using serum samples, which are subjected to centrifugation. The analysis employs a direct chemiluminescent method based on a competitive immunoassay using products from Siemens. The Atellica IM VitD assay utilizes paramagnetic particles (PMP) that are covalently bonded to fluorescein-labeled mouse monoclonal antibodies, acridinium ester-labeled mouse monoclonal antibodies specific to 25(OH) Vitamin D, and fluorescein-labeled Vitamin D in a competitive immunoassay format. Calibration is conducted periodically, and quality control is performed daily.

### 2.4. Genotyping

Whole blood samples (3 mL) were collected in EDTA tubes, and genomic DNA was isolated and genotyped at Theragen Health Co., Ltd., a genetic testing institution officially designated by the Korea Disease Control and Prevention Agency (KDCA) as a specialized non-clinical testing facility. Genotyping was performed using the Axiom™ DNA microarray platform in accordance with standard DNA preparation protocols.

Purified DNA was fragmented into 25–125 bp segments, resuspended, and hybridized to the Theragen Precision Medicine Research Array (Theragen PMRA), a customized array derived from the Asian Precision Medicine Research Array (Thermo Fisher Scientific, Waltham, MA, USA). After hybridization, stringent washing procedures were applied to remove nonspecific background signals and reduce noise from random ligation events. Genotype calling was then conducted following the manufacturer’s instructions, yielding data for 699,670 single nucleotide polymorphisms (SNPs) with comprehensive genome-wide coverage across five major population groups.

All samples underwent rigorous quality control procedures. Only datasets with a dish quality control (DQC) value > 0.82 and a sample call rate > 0.95 were retained for downstream analyses to ensure high reliability and accuracy. From the final genome-wide SNP dataset, polymorphisms located in SOST, LRP5, and ESR1—genes with well-established relevance to osteoporosis susceptibility—were extracted for further investigation.

### 2.5. Statistical Analysis

All statistical analyses were conducted using IBM SPSS Statistics version 29.0.2.0 (IBM Corp., Armonk, NY, USA). Differences in baseline characteristics between groups were evaluated using chi-square tests for categorical variables and ANOVA for continuous variables. We assessed additive model associations between genetic polymorphisms and osteoporosis prevalence and incidence as primary outcomes, and osteoporosis-related phenotypes as secondary outcomes, using logistic regression models for binary outcomes and linear regression models for continuous outcomes. Statistical significance was defined as *p* < 0.05. Given that three SNPs were tested for each phenotype, a Bonferroni-adjusted significance threshold of *p* < 0.016 was applied to account for multiple comparisons.

## 3. Results

### 3.1. Population Characteristics

The characteristics of the study participants are presented in Table 2.

### 3.2. Genotype Distribution

The genotype frequencies of SOST, LRP5, and ESR1 polymorphisms are summarized in Table 3. Across all three polymorphisms (SOST, LRP5, and ESR1), genotype distributions were broadly comparable between men and women. The TC genotype for SOST, CC genotype for LRP5, and GG genotype for ESR1 were the most prevalent across the study population. The genotype calling clusters from the experimental data can be found in Supplementary Figure S1.

#### 3.2.1. Association with Osteoporosis or Vitamin D

The association analyses between the three genetic single nucleotide polymorphisms (SNPs) (SOST_rs1513670, LRP5_rs3736228, and ESR1_rs6929137) and osteoporosis status showed no statistically significant relationships (Table 4). In contrast, the analysis of serum Vitamin D concentration revealed a significant association with the SOST polymorphism, whereas LRP5 and ESR1 showed no significant relationships (Table 5). SOST was strongly associated with serum Vitamin D levels (β ± s.e. = −4.836 ± 0.992, *p* = 1.7 × 10^−6^), indicating that individuals with the risk genotype exhibited substantially lower Vitamin D concentrations.

#### 3.2.2. Population Characteristics by SOST Genotype

Across the three SOST SNP genotypes, most anthropometric, lifestyle, and clinical characteristics were generally comparable. However, a notable finding was the markedly lower serum Vitamin D levels in carriers of the TC and CC genotypes, consistent with the statistical association previously identified (Table 6).

## 4. Discussion

In this study, we tested three genetic polymorphisms obtained from routine health examinations by genetic testing. We investigated the associations between key osteoporosis-related genetic polymorphisms—SOST_rs1513670, LRP5_rs3736228, and ESR1_rs6929137—and osteoporosis prevalence as well as biochemical markers relevant to bone metabolism. Although none of the examined variants showed significant associations with osteoporosis status, we identified a statistically significant relationship between the SOST polymorphism and serum vitamin D concentrations. Individuals with carriers of the TC and CC genotypes exhibiting substantially lower vitamin D levels compared with TT carriers. These results provide hypothesis-generating finding between WNT signaling and vitamin D metabolism, even in the absence of direct effects on osteoporosis diagnosis itself [25].

The lack of association between SOST, LRP5, and ESR1 SNPs and osteoporosis status is partly consistent with prior studies reporting heterogeneous and context-dependent effects of these polymorphisms [26,27,28]. Although the WNT signaling pathway—particularly LRP5 and SOST (encoding sclerostin)—plays a central role in osteoblast differentiation and bone formation, many genetic effects on osteoporosis are known to be modest and influenced by age, hormonal status, lifestyle factors, and gene–environment interactions [29,30]. Similarly, variants in ESR1 have shown strong relevance in postmenopausal women in certain populations, but their effects are not always detected in mixed-sex or middle-aged cohorts such as ours [31]. The relatively low prevalence of osteoporosis and fractures in this population may also limit statistical power to detect genotype associations with clinical outcomes.

In contrast, the significant association between SOST polymorphisms and serum vitamin D levels is biologically plausible and merits careful consideration [32]. Sclerostin, the protein encoded by SOST, is a key inhibitor of WNT/β-catenin signaling and is strongly regulated by hormonal, inflammatory, and nutritional factors [33]. Vitamin D, through its receptor-mediated genomic actions, modulates osteoblast activity, calcium homeostasis, and bone turnover—processes that are closely interconnected with WNT signaling [25]. Experimental studies have suggested that vitamin D may suppress SOST expression, whereas impaired WNT pathway activity may influence vitamin D metabolism or receptor signaling [33].

Beyond mechanistic plausibility, our findings suggest potential biological implications of the observed association between SOST polymorphisms and serum vitamin D levels. Vitamin D deficiency is one of the most common modifiable risk factors for poor bone health, and its interaction with WNT pathway regulation has been increasingly recognized as an important component of bone metabolism [33]. In this context, the association observed in the present study may reflect a biologically meaningful link between WNT signaling and vitamin D metabolism, even in the absence of a direct association with osteoporosis status. It is also noteworthy that anthropometric characteristics, lifestyle factors (including smoking, alcohol intake, and exercise), and most biochemical variables were comparable across SOST genotype groups, suggesting that the observed differences in serum vitamin D levels are unlikely to be attributable to major behavioral or metabolic confounders. This supports the possibility of a genotype-related effect on vitamin D status.

Previous studies have consistently reported a high prevalence of vitamin D deficiency in the Korean population. Nationwide analyses have shown low serum 25-hydroxyvitamin D [25(OH)D] concentrations across multiple age groups, with marked variations by sex and season [34]. Similarly, Yu et al. demonstrated that vitamin D deficiency and insufficiency are common among Korean adults, particularly during winter and spring, even among ostensibly healthy individuals. In this population-specific context, the relatively low serum 25(OH)D levels observed in the present study may therefore reflect underlying characteristics of the Korean population rather than measurement error [35]. Nevertheless, given the lack of detailed information on vitamin D supplementation, sun exposure, and seasonal timing of sample collection, these findings should be interpreted with caution. Although the SOST variants did not translate into a higher prevalence of osteoporosis in this study, the documented reduction in serum vitamin D levels among TC/CC carriers may still be relevant to long-term skeletal health, particularly as individuals age or transition into menopause [36]. However, in the absence of predictive performance analyses and comparisons with established clinical risk factors, the present findings should be regarded as hypothesis-generating rather than indicative of immediate clinical applicability.

Several limitations of this study should be acknowledged. First, osteoporosis was defined using a combination of bone mineral density (BMD) criteria and osteoporosis-related diagnostic codes (KCD-8: M80–M85). Although this approach enabled the inclusion of longitudinal real-world clinical data, the use of administrative diagnostic codes may introduce phenotype heterogeneity, as these codes encompass multiple osteoporosis-related conditions and may not always precisely correspond to BMD-based diagnoses. In addition, BMD data were not available for all individuals assigned osteoporosis-related diagnostic codes, which may have resulted in misclassification in a subset of participants. Second, information on lifestyle and environmental factors known to influence bone health—including vitamin D supplementation, calcium intake, sun exposure duration, and seasonal variation at the time of sample collection—was not available. These variables are difficult to capture accurately in retrospective, large-scale clinical and genetic datasets and therefore could not be incorporated into the present analysis. Although these factors are discussed given their established importance in the Korean population, the lack of detailed exposure data limits our ability to fully account for their potential confounding effects.

Despite these limitations, the study provides meaningful evidence that SOST polymorphisms may influence serum vitamin D levels, highlighting a potentially important biological link that warrants further investigation. Future research should examine whether this relationship is mediated by altered sclerostin expression or downstream WNT pathway activity, and whether genotype-dependent differences in vitamin D contribute to changes in BMD or fracture risk over time. Replication in larger, age-stratified cohorts—including postmenopausal women and elderly adults—would be particularly valuable.

## 5. Conclusions

In conclusion, while SOST, LRP5, and ESR1 polymorphisms were not directly associated with osteoporosis in this population, the strong relationship between the SOST variant and reduced vitamin D concentrations underscores the importance of genetic regulation of bone metabolism beyond classical clinical endpoints. These findings add to the growing body of evidence linking WNT signaling with vitamin D pathways and may provide a foundation for personalized bone-health strategies informed by genetic background.

## Figures and Tables

**Table 1:** References for osteoporosis-related targets.

Candidate Genes	Description	Target SNP	Chr	bp (hg19)	Locus	Effect Allele	Reference	Association	Population
SOST	Sclerostin	rs1513670	17	41807331	3′ Downstream	T allele	[18]	Decreased Bone mineral density	European
						T allele	[19]	Decreased Bone mideral density	Chinese
LRP5	Low-density lipoprotein receptor-related protein 5	rs3736228	11	68201295	LRP5 missence	C allele	[20]	Low Mineral Density	Romanian
						C allele	[21]	Increase Rheumatoid Arthritis	Pakistan
						C allele	[22]	Low Calcaneal ultrasound parameter	Caucasian
ESR1	Estrogen Receptor 1	rs6929137	6	151936677	5′ Upstream	A allele	[23]	Effect size not reported	European
						A allele	[18]	Decreased BMD	European

**Table 2:** Population characteristics.

Variables	Total	Male	Female	*p*-Value
n	843	461	382	
Age (Years Old)	49.6 ± 11.5	49.8 ± 11.2	49.3 ± 11.8	0.981
Smoking Habit (n, %)				
No	613 (72.7)	248 (53.8)	365 (95.5)	<0.001
Quit	120 (14.2)	111 (24.1)	9 (2.4)	
Current	110 (13.0)	102 (22.1)	8 (2.1)	
Alcohol Drinking (n, %)				
No	403 (47.8)	141 (30.6)	262 (68.6)	<0.001
Quit	11 (1.3)	8 (1.7)	3 (0.8)	
Current	429 (50.9)	312 (67.7)	117 (30.6)	
Regular Exercise (n, %)				
No	301 (35.7)	154 (33.4)	147 (38.5)	0.238
Yes	542 (64.3)	307 (66.6)	235 (61.5)	
Height (cm)	167.5 ± 8.7	173.5 ± 5.7	160.5 ± 5.8	<0.001
Weight (kg)	66.3 ± 13.6	74.6 ± 11.4	56.5 ± 8.6	<0.001
Body Mass Index (kg/m^2^)	23.5 ± 3.5	24.7 ± 3.2	21.9 ± 3.3	<0.001
Serum Calcium (mg/dL)	9.4 ± 0.4	9.4 ± 0.4	9.3 ± 0.4	0.980
Serum Vitamin D, 25(OH)D (nmol/L)	27.8 ± 12.7	26.9 ± 11.6	28.5 ± 13.4	<0.001
Osteoporosis case All (n, %)	70 (8.3)	11 (2.4)	59 (15.4)	<0.001
Newly Developed Osteoporosis case (n, %)	29 (3.4)	6 (1.3)	23 (6.0)	<0.001
Fracture (n, %)	1 (0.1)	1 (0.2)	0 (0)	0.790

Note. *p*-values were calculated using chi-square tests or *t*-tests to evaluate sex-specific differences.

**Table 3:** Genotype distribution.

Gene (SNP)	chr:bp	Alleles		Alt. Allele Frequencies		HWE		Group	Genotype Frequencies, n (%)		
		Ref.	Alt.	1000G EAS	This Study	χ^2^	*p*-Value		Ref/Ref	Ref/Alt	Alt/Alt
SOST (rs1513670)	17:41807331	T	C	0.45	0.40	10.7	>0.005	Total	330 (39%)	358 (42%)	155 (18%)
								Male	197 (43%)	186 (40%)	78 (17%)
								Female	133 (35%)	172 (45%)	77 (20%)
LRP5 (rs3736228)	11:68201295	C	T	0.24	0.24	1.8	>0.2	Total	488 (58%)	298 (35%)	57 (7%)
								Male	286 (62%)	141 (31%)	34 (7%)
								Female	202 (53%)	157 (41%)	23 (6%)
ESR1 (rs6929137)	6:151936677	G	A	0.28	0.29	1.7	>0.2	Total	418 (50%)	356 (42%)	639 (8%)
								Male	222 (48%)	205 (44%)	34 (7%)
								Female	196 (51%)	151 (40%)	35 (9%)

**Table 4:** Genetic association results of osteoporosis prevalence and incidence with controlling age, sex, BMI, drink, smoke, and exercise.

SNPs	Osteoporosis Case All			Newly Developed Osteoporosis Case		
	Odds Ratio	95% CI	*p*-Value	Odds Ratio	95% CI	*p*-Value
SOST (rs1513670)	1.06	0.744–1.151	0.748	0.865	0.510–1.468	0.592
LRP5 (rs3736228)	1.126	0.755–1.680	0.559	1.088	0.595–1.988	0.784
ESR1 (rs6929137)	0.902	0.613–1.326	0.599	1.247	0.713–2.180	0.438

**Table 5:** Genetic association results of 25(OH)D with controlling age, sex, BMI, drink, smoke, and exercise.

SNPs	25(OH)D		
	Beta	Se	*p*-Value
SOST (rs1513670)	−4.836	0.992	1.7 × 10^−6^
LRP5 (rs3736228)	0.391	1.211	0.747
ESR1 (rs6929137)	1.861	1.178	0.115

**Table 6:** Population Characteristics Among The SOST Genotypes.

Variables	SOST rs1513670 Genotypes			*p*-Value
	TT	TC	CC	
n	330	358	155	
Sex (female, %)	40.3	48	49.7	0.060
Age (Years Old)	49.9 ± 10.9	50.1 ± 11.1	48.8 ± 12.5	0.446
Smoking Habit (n, %)				0.203
No	233 (70.6)	263 (73.5)	117 (75.5)	
Quit	54 (16.4)	42 (11.7)	24 (15.5)	
Current	43 (13.0)	53 (14.8)	14 (9.0)	
Alcohol Drinking (n, %)				0.200
No	142 (43.0)	178 (49.7)	83 (53.5)	
Quit	5 (1.5)	5 (1.4)	1 (0.6)	
Current	183 (55.5)	175 (48.9)	71 (45.8)	
Regular Exercise (n, %)				0.321
No	111 (33.6)	127 (35.5)	63 (40.6)	
Yes	219 (66.4)	231 (64.5)	92 (59.4)	
Body Mass Index (kg/m^2^)	23.5 ± 3.5	23.3 ± 3.5	23.6 ± 3.6	0.657
Serum Calcium (mg/dL)	9.4 ± 0.3	9.4 ± 0.4	9.4 ± 0.4	0.771
Serum Vitamin D, 25(OH)D (nmol/L)	32.9 ± 14.9	25.8 ± 13.11	24.59 ± 10.4	<0.001
Osteoporosis case (%)	8.5	10.3	12.3	0.379
Fracture (%)	0	0.3	0	0.508
Type 2 Diabetes case (%)	13.9	13.7	10.3	0.508
Hypertension case (%)	16.1	10.3	11	0.061

## Data Availability

The data that support the findings of this study are available from Seoul St Mary’s hospital. Restrictions apply to the availability of these data, which were used under license for this study.

## Supplementary Materials

The following supporting information can be downloaded at: https://www.mdpi.com/article/10.3390/life16020259/s1, Figure S1. Genotype cluster plots for the study samples.

## Abbreviations

The following abbreviations are used in this manuscript:

BMD	Bone Mineral Density
SMH	Seoul St. Mary’s Hospital
KCDA	Korea Disease Control and Prevention Agency
SNPs	Single Nucleotide Polymorphisms
DQC	Dish Quality Control
