# Supplementary figures and images for: Real-World Assessment of Osteoporosis-Related Polymorphisms: Negative Findings for Osteoporosis and an Exploratory Association with Vitamin D
Source: Life (Basel). 2026 Feb 3;16(2):259. doi: 10.3390/life16020259 (PMC12942187; doi:10.3390/life16020259)

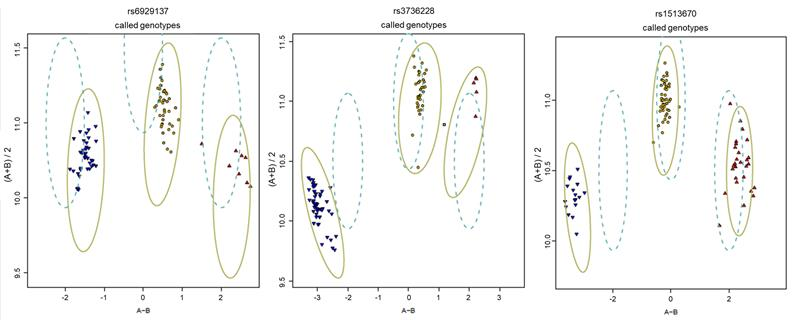

Supplement: Supplementary file 1 [file life-16-00259-s001.zip › life-4086199-supplementary.tif]
